# Synergies for Improving Oil Palm Production and Forest Conservation in Floodplain Landscapes

**DOI:** 10.1371/journal.pone.0095388

**Published:** 2014-06-02

**Authors:** Nicola K. Abram, Panteleimon Xofis, Joseph Tzanopoulos, Douglas C. MacMillan, Marc Ancrenaz, Robin Chung, Lucy Peter, Robert Ong, Isabelle Lackman, Benoit Goossens, Laurentius Ambu, Andrew T. Knight

**Affiliations:** 1 Durrell Institute for Conservation and Ecology, School of Anthropology and Conservation, University of Kent, Canterbury, Kent, United Kingdom; 2 HUTAN/Kinabatangan Orang-utan Conservation Programme, Kota Kinabalu, Sabah, Malaysia; 3 Living Landscape Alliance, Reading, Berkshire, United Kingdom; 4 Borneo Futures initiative, People and Nature Consulting International, Jakarta, Indonesia; 5 Technological Education Institute of Kavala, Department of Forestry and Management of Natural Environment, Drama, Greece; 6 North England Zoological Society, Chester, Cheshire, United Kingdom; 7 C H Williams Talhar and Wong (Sabah) Sdn Bhd, Sandakan, Sabah, Malaysia; 8 Danau Girang Field Centre, c/o Sabah Wildlife Department, Kota Kinabalu, Sabah, Malaysia; 9 Forest Research Centre, Sabah Forestry Department, Sandakan, Sabah, Malaysia; 10 Organisms and Environment Division, School of Biosciences, Cardiff University, Cardiff, Wales, United Kingdom; 11 Sabah Wildlife Department, Kota Kinabalu, Sabah, Malaysia; 12 Department of Life Sciences, Imperial College London, Ascot, Berkshire, United Kingdom; 13 Department of Botany, Nelson Mandela Metrropolitan University, Port Elizabeth, Eastern Cape, South Africa; Institute of Agronomy, University of Lisbon, Portugal

## Abstract

Lowland tropical forests are increasingly threatened with conversion to oil palm as global demand and high profit drives crop expansion throughout the world’s tropical regions. Yet, landscapes are not homogeneous and regional constraints dictate land suitability for this crop. We conducted a regional study to investigate spatial and economic components of forest conversion to oil palm within a tropical floodplain in the Lower Kinabatangan, Sabah, Malaysian Borneo. The Kinabatangan ecosystem harbours significant biodiversity with globally threatened species but has suffered forest loss and fragmentation. We mapped the oil palm and forested landscapes (using object-based-image analysis, classification and regression tree analysis and on-screen digitising of high-resolution imagery) and undertook economic modelling. Within the study region (520,269 ha), 250,617 ha is cultivated with oil palm with 77% having high Net-Present-Value (NPV) estimates ($413/ha**^−^** ^yr^–$637/ha**^−^** ^yr^); but 20.5% is under-producing. In fact 6.3% (15,810 ha) of oil palm is commercially redundant (with negative NPV of $-299/ha**^−^** ^yr^-$-65/ha**^−^** ^yr^) due to palm mortality from flood inundation. These areas would have been important riparian or flooded forest types. Moreover, 30,173 ha of unprotected forest remain and despite its value for connectivity and biodiversity 64% is allocated for future oil palm. However, we estimate that at minimum 54% of these forests are unsuitable for this crop due to inundation events. If conversion to oil palm occurs, we predict a further 16,207 ha will become commercially redundant. This means that over 32,000 ha of forest within the floodplain would have been converted for little or no financial gain yet with significant cost to the ecosystem. Our findings have globally relevant implications for similar floodplain landscapes undergoing forest transformation to agriculture such as oil palm. Understanding landscape level constraints to this crop, and transferring these into policy and practice, may provide conservation and economic opportunities within these seemingly high opportunity cost landscapes.

## Introduction

Lowland tropical forest ecosystems contain some of the highest levels of species endemism and biological diversity globally [1], [2]. However, tropical lowland forests are under increasing risk of conversion to oil palm (*Elaeis guineensis*) which drives biodiversity loss [2] and compromises forest functioning and maintenance of vital ecosystem services [3], [4], [5]. In 2012 over 17.1 million hectares of permanent cultivated cropland worldwide was comprised of oil palm agriculture compared with 9.97 million hectares in the year 2000 [6]. Oil palm establishment continues to increase within lowland areas of Malaysia and Indonesia, the two dominant producing countries, but also elsewhere in tropical Asia, Africa and central and South America [6], [7]. Brazil alone has 2 million hectares of lowland forest identified as suitable for oil palm, and similarly the Democratic Republic of Congo, Indonesia, Peru and Columbia have an estimated 0.78 million, 0.61 million, 0.46 million and 0.42 million hectares respectively, of forest deemed as suitable for oil palm [8]. Expansion of this crop is largely due to its high opportunity costs driven by accelerating global demand for this versatile oil coupled with high palm production (i.e. yield) capacity [9].

Oil palm plantations are limited to low elevation areas and are in direct conflict with tropical lowland forests, including those found within riverine floodplains. Riverine floodplains are characterised by high levels of biodiversity and productivity [10]. These regions are low lying and subject to periodic inundation by associated rivers or streams [11]. Consequently, these landscapes present heterogenous environmental characteristics that dictate variable land suitability for oil palm cultivation, with unsuitable areas principally linked to seasonal and/or tidal inundation events. The palm *Elaeis guineensis* is flood intolerant with mortality occurring from root rot within two weeks of ground saturation and/or low oxygen levels or from saline water [12]. Palm mortalities reduce plantation yields and in-turn financial returns [13]. Nevertheless, simplistic biophysical criteria are often used by governments and agencies for agricultural zoning for oil palm that includes slope (<25°), elevation (<500 m) and soil types within suitable climatic zones [14]. These criteria may fail to capture regionalised constraints for this crop.

We investigated forest and oil palm dynamics within a floodplain system in the Malaysian context. In Malaysia it is estimated that between 1990–2005, 55–59% of Malaysia’s oil palm extent replaced old-growth and secondary forests [15]. In Malaysia, Sabah (Borneo) is the largest oil palm planted state contributing 28.6% of the country’s total oil palm extent [16]. In 2011, 1.43 million hectares (19.3%) of Sabah’s terrestrial extent was under oil palm which could increase up to 2.1 million hectares by 2025, depending on land suitability [16], [17]. It is likely that oil palm expansion will continue to target the eastern State floodplains areas that have very high yield potential. We employed the Lower Kinabatangan landscape, the largest floodplain in Sabah, as a ‘model system’ to understand spatial and economic components of forest conversion to oil palm in a riverine floodplain. We highlight the need for improving biophysical criteria used by governments in agricultural zoning for oil palm and emphasise the need for regional land-use planning of floodplain landscapes to promote synergies between agricultural development and biodiversity and ecosystem conservation goals. Understanding landscape level constraints to this crop, and transferring these into policy and practise, may provide conservation opportunities within these seemingly high opportunity cost landscapes.

## Materials and Methods

### Study Area

**Figure 1:**
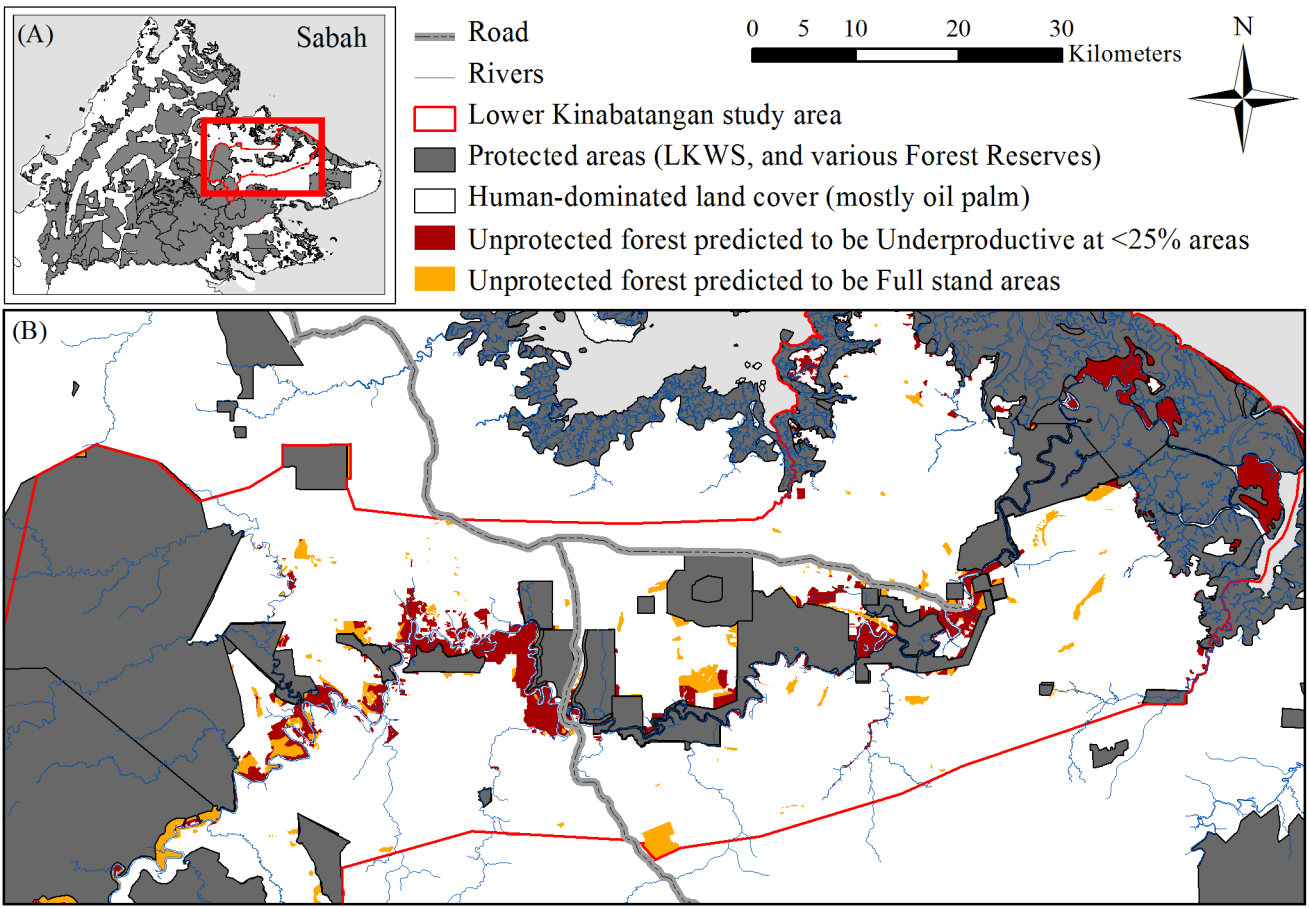
Study region and result from the CART analysis. (A) Map of the study region, the Lower Kinabatangan (red outline) within the context of Sabah, Malaysian Borneo. Protected areas (including all ‘use’ forests) are in grey. (B) Map of the Lower Kinabatangan study region with the spatial output from the Classification and Regression Tree analysis (CART) overlaid with the unprotected forest areas. Dark red demonstrates areas predicted as unsuitable for oil palm (i.e. areas that would be <25% palm capacity and commercially redundant); orange areas are predicted for full stands of oil palm (i.e. suitable areas).

The study area comprised 520,269 ha of the Lower Kinabatangan floodplain region, in eastern Sabah (Figure 1(A)). The region experiences mean monthly temperatures of 21–34°C and average annual rainfall of 3,000 mm [18]. Forest types encompass those associated with mangrove, flooded forest and dry (humid) forest systems, which are threatened forest types [19]. Remaining forests have been heavily impacted from past commercial timber exploitation and extensive forest conversion resulting in significant forest loss, severe fragmentation and degradation within the forest ecosystem [20], [21]. Yet, these forests remain important habitat for biodiversity harbouring 129 species of mammal, 314 species of birds, 101 species of reptiles, and 33 species of amphibians [22]. Many of these species are threatened IUCN (International Union for Conservation of Nature) Red List species such as the Bornean elephant [23], Bornean orangutan [24] and proboscis monkey [25]. These forests provide species habitat, are instrumental in connecting (at least in part) the fragmented protected areas network; as well as crucial for facilitating multiple ecological processes needed for the functioning of this ecosystem. Attempts to safeguard remaining forests has led to the gazetting of the Lower Kinabatangan Wildlife Sanctuary (LKWS, at 27,960 ha) in 2005. Other protected areas include protection forest reserves and virgin jungle forest reserves. Use-forests include the mangrove forest reserves and commercial (i.e. production) forest reserves; all herein referred to as protected areas (PA’s). However, significant areas of unprotected forest remain outside the protected areas or production forests and on State land or land ‘alienated’ land for agriculture. Alienated land is leased (long-term) State land, under title and granted for a specific purpose such as oil palm [26]. Despite over a decade of conservation initiatives to secure these unprotected forests for conservation purposes they remain threatened largely with conversion to oil palm.

### Forest Extent and Mapping Forest Types

Forest vegetation types were identified using SPOT5 10 m satellite images captured on 25/11/2007 and two on 19/06/2009; as well as two Landsat TM5 30 m images captured on 27/07/2006 and 11/08/2009 (downloaded from www.usgs.gov). Images were orthorectified and registered and indices calculated for: Normalized Difference Vegetation Index (NDVI); Normalized Difference Moisture Index (NDMI); and Soil Adjusted Vegetation Index (SAVI). Spectral transformations were performed including reflectance for SPOT5 and Tasseled Cap transformation on the Landsat TM5 Images [27]. We used a step-wise Object-Based Image Analysis (OBIA in eCognition Developer 8.7) for the classification that creates meaningful ‘objects’ by segmenting images into groups of spectrally similar pixels and spatial characteristics [28]. The eastern and western Kinagantagan areas were classified separately because different image capture dates prohibited the development of a common classification rule set. For training the classifier and testing the result, 1,938 ground validation points were collected in-field using a stratified random sample. The stratification was conducted on dominant regional forest types idenftified by local experts (for details see Table 1). Within a 20 m buffer around each validation point, data were recorded on: habitat type; degradation level (low, medium, high); percentage canopy cover; and dominant tree species with diameter at breast height (DBH) class. The ground truthed dataset was halved, one portion for training the classifier and the other for testing the result.

**Table 1:** Forest system and forest type classes found in the Kinabatangan with flooding periods and extents in hectares (ha) as calculated from the Object-Based Image Analysis classification.

Forest systems and forest type class	Annual flood-ing period	Total forest	Protected forest	Unprot-ected forest
		ha (%)	ha (%)	ha (%)
Mangrove forest:				
*Beach forest:* Occurs on sandy substrate alongcoastal areas. Dominant species includes*Casuarina equisetifolia.*	Tidal	5,327 (2)	4,672 (2)	655 (2)
*Mangrove forest:* Found in saline coastal sediments.Dominant species include *Rhizophora apiculata.*	Tidal	12,863 (5)	12,357 (6)	506 (2)
*Nipah palm forest:* Native type of palm (*Nypa* *fruticans*) found within the mangrove system eitherin mono-stands or mixed with *Rhizophora apiculata*	Tidal	26,618 (11)	25,399 (11)	1,219 (4)
*Transitional forest:* Occurs between mangrove andfreshwater swamp forest. Brackish water. Dominantspecies of *Heritiera littoralis, Ilex cymosa, Excoecaria* *agallocha.*	Semi-tidal	13,849 (6)	10,567 (5)	3,282 (11)
*Seasonally flooded forest:*				
*Freshwater swamp forest:* Formed in backswampsand largely on poorly drained soil. Common speciesinclude *Dillenia excelsa, Croton oblongus,* *Mallotus muticus.*	>6 mths	22,284 (9)	16,721 (8)	5,563 (18)
*Seasonal freshwater swamp forest:* Heavy degradationthought to have occurred with many pioneer species.Common species include *Macaranga gigantea,* *Pterospermum elongatum, Cananga odarata.*	3–6 mths	12,501 (5)	8,253 (4)	4,248 (14)
*Peat swamp forest:* Oligotropic peat substrate, poorlydrained forests exposed to flooding.Common treespecies include *Lophopetalum multinerviu, Baccaurea*,*Campnosperma coriaceum*,*Syzygium* and *Anisoptera costata*.	>6 mths	2,132 (1)	2,102 (1)	30 (<0)
*Swamp:* Open reed, swampvegetation. Dominant tree species *Excoecaria indica*	>9 mths	2,750 (1)	2,048 (1)	702 (2)
*Lowland dry forest:*				
*Lowland dry forest:* Previous dipterocarp forest,secondary forest with species including, *Nauclea* *subdita, Neolamarckia cadamba, Glochidion rubrum*	<3 mths	39,008 (16)	30,693 (14)	8,315 (28)
*Lowland dry dipterocarp forest:* Logged lowlandmixed dipterocarp forest, dominated with *Dipterocarp* *sp.*	Never/Rarely	101,878 (41)	100,866 (46)	1,012 (3)
*Limestone forest:* Gomantong substrate association ofhill and ridge escarpments. Low human disturbance.Dominant species include *Dryobalanops lanceolata*,*Shorea pauciflora*, *Parashorea* *malaanonan* and *Dipterocarpus caudiferus.*	Never/Rarely	1,679 (1)	1,392 (1)	287 (1)
*Mixed vegetation types:*				
*Severely degraded:* areas of severedegradation with unknown previousforest types dominated byshrub/low lying vegetation.	Varied	10,511 (4)	6,159 (3)	4,352 (14)
Sum of hectares		251,400	436,295	30,173

For image segmentation and object generation within eCognition, SPOT5 10 m resolution data were employed for delimitation of ‘objects’ within the images. Following trialing of numerous scale parameters, a scale of 15 was adopted. Colour/Shape and smoothness/compactness were set (0.9/0.1 and 0.5/0.5 respectively) [29]. All spatial data were used for ruleset development and the identification of features with the highest discrimination ability. The Support Vector Machine algorithm (SVM) within eCognition was employed to run the classification because it performed better in reproducing the training set than other trialled algorithms (i.e. Byes or classification and regression tree) [30], [31]. We used a generic forest extent auxiliary layer, digitised from 2.5 m SPOT5 satellite images captured 12/12/2010 and 01/06/2011, to restrict the classification to areas of known forest (these images were not used in the classification due to inappropriate bands). Despite the high accuracy of the SVM classifications, classification refinement was undertaken on selected known misclassified objects using ground knowledge or neighborhood based features.

The eastern and western classifications were exported as shapefiles and ‘merged’ to form one continuous layer. An accuracy assessment was performed using an error confusion matrix method using the second portion of the ground truth dataset, and overall classification accuracy and kappa statistics were calculated for both portions [29].

### Mapping Existing Oil Palm

**Figure 2:**
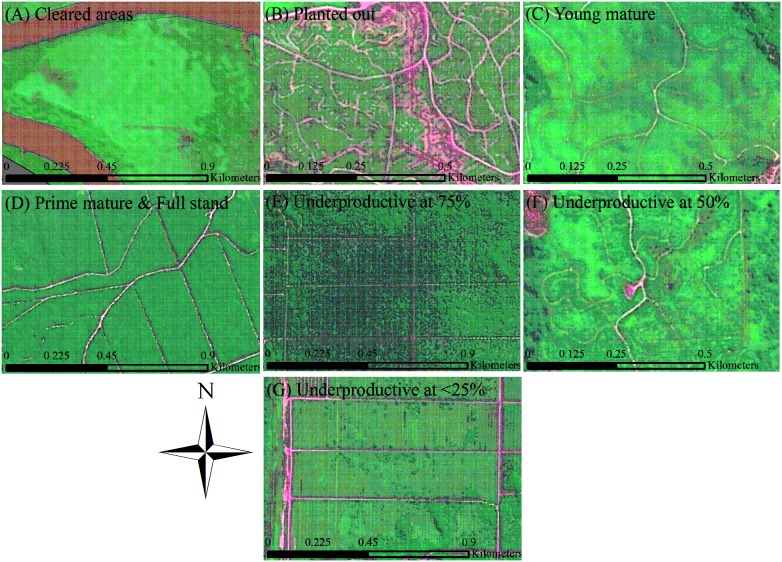
Examples of oil palm age and productivity classes. Images (A) to (D) show examples of the four age classes used for the oil palm mapping; and, images (E) to (G) show examples of underproductive classes, using the 2.5 m SPOT satellite images.

Oil palm age and productivity classes were digitised using the 2.5 m SPOT5 images. Age classes were defined by characteristics viewable in the images that were associated with planting preparation and/or age associated features (see Table 2 for descriptions and Figure 2 for examples). To inform the visual characteristics, we used two geo-referenced maps (in ARcGIS 10) from large oil palm estates within the region (Sime Darby and Hilco Estates) that contained planting years for each oil palm ‘block’. For productivity classes we based these on palm capacity. Optimal planting density for oil palm typically ranges from 128 to 148 palms per hectare depending on factors such as genotypes, environmental conditions, soil types and management practises [32]. In our study region 136 stands (i.e. palms) per hectare (SPH) is used, as recommended by the Malaysian Palm Oil Board (MPOB) and we used 136 SPH as our upper limit value for palm capacity (i.e., 136 SPH = 100% palm capacity). We classified the underproductive classes by defining the range of palm capacity within each class. Descriptions of each class can be seen in Table 2 and examples in Figure 2. In brief however, our oil palm categories were: (1) Cleared areas; (2) Planted out (0–2 years old); (3) Young mature (3–6 years, and ranging from 76–100% palm capacity); and (4) Prime mature at ‘Full stand’ (7–24 years, and ranging from 76–100% palm capacity); (5) Underproductive at 75% (areas that ranged from 51%–75% SPH capacity); (6) Underproductive at 50% (areas that had approximately 26–50% SPH capacity); and (7) Underproductive at ≤25% (areas that had approximately 25% SPH capacity or less).

**Table 2:** Oil palm age and productivity classes and descriptions, mapped using SPOT5 2.5

Oil palm classes	Description
Cleared areas	Areas cleared of forest cover with bare earth or grass like vegetation cover. No roads laid nor ground prepared for oil palm planting (Figure 2A).
Planted out	Areas had roads cut and laid, ground prepared for planting, and in some areas palms planted out (palms would be approximately 2 years or younger) (Figure 2B).
Young mature	Young mature palms were visible but palm fronds did not overlap. Low-lying (leguminous) vegetation was visible. Palms in this category range from 3–6 years (Figure 2C).
Prime mature and Full stand	Palms in prime yield (7–24 years) and ‘Full stand’ (i.e., 76–100% palm capacity). Class had closed canopies i.e., overlapping fronds of neighbouring plants. Canopy closes at 7 years in areas with 136 palms per ha. Homogeneous texture (Figure 2D).
Underproductive at 75%	Class ranged from 51%–75% palm capacity. Class included: older palm (where mortality would naturally start to occur) or areas prone to water logging (dimpling effect can be seen in blocks) (Figure 2E).
Underproductive at 50%	Class ranged from 26–50% palms per ha capacity and were sometimes on slightly undulating areas or areas with water logging/flooding issues (Figure 2F).
Underproductive at 25%	Class had ≤25% palms per ha and were largely associated with areas that experience annual flooding and/or daily inundation from tides (Figure 2G).

Classification accuracy was determined through remotely obtained information as no ground-data was available. We used all recent (16/09/2009 and 24/08/2011) high-resolution tiles from Google Earth images (see http://earth.google.com) in our region to validate our oil palm age and productivity map. Although our satellite imagery was 2.5 m, which allowed individual palms to be seen, the higher resolution tiles in Google Earth enabled better interpretation ability and therefore permitted the validation of classes. We defined these tiles within a GIS (ArcGIS 10), generated 120 random points (see Figure S1 in File S1) with assigned unique identity numbers. We imported the validation points into Google Earth, assessed and allocated an oil palm class for each point. An error confusion matrix method was used to assess map accuracy and kappa statistic was calculated [29].

### Economic Models for Oil Palm

We developed economic models to calculate the net present value (NPV) of oil palm for four oil palm class scenarios (see Tables S1 (a+b) to S4 (a+b) in File S2). These classes included; (1) ‘Full stand’ areas which incorporated Young mature and Prime mature oil palm classes that ranged from 76% (i.e., 103 SPH) to 100% (based on a maximum of 136 SPH) palm capacity (and combined enumerated 96.3% of this class extent). We also included Cleared areas and Planted out classes into ‘full stand’ under the assumption they will fall into this palm capacity range in the future or once planted (collectively these classes made up only 3.7% of the ‘full stand’ class extent); (2) ‘Underproductive at 75%’ which assumed 51% (69 SPH) to 75% (102 SPH) palm capacity; (3) ‘Underproductive at 50%’ at 26% (35 SPH) to 50% (68 SPH) palm capacity; and (4) ‘Underproductive at ≤25%’ which ranged from 0% (0 SPH) to 25% (34 SPH) palm capacity. We modelled the NPV of the lowest and highest palm capacity percentage range for the four classes.

Our economic models assumed a typical 25 year crop life as per industry standard [16]. For each model a yield curve was used spanning the 25 year time frame (see oil palm yields in Figure S2 in File S2). Yields vary considerably over the lifespan of the crop with declines occurring after peak years due to factors such as palm mortality. To generate our yield estimates we used data based on actual yields from the region for full stand areas of 136 SPH (obtained from C H Williams). For the ‘Full stand’ at 136 SPH (100% palm capacity) NPV model, we used a maximum yield of 30 metric tons (t) of fresh fruit bunches (FFB) per hectare (ha) in peak years (years 8–11) declining to 17 t/FFB/ha in year 25, averaging at 21.92 t/FFB^−25^. For all other models, we vary annual yields through the 25 year model timeframe, by calculating the proportion of yield (in t/FFB/ha) needed against our maximum ‘Full stand’ (100% at 136 SPH) yield values. In our models we assume a direct relationship between the number of SPH (or percentage thereof) and yield. We discuss the pitfalls and limitations of these assumptions in the discussion.

Within our models we retained FFB as our revenue unit rather than converting to crude palm oil (CPO) as for smallholders and commercial estates with no processing mills, revenue is derived from the sale of FFB. We based our model largely around 2011 data. As a result we used a constant FFB price of US$178 t/FFB, based on average 2011 values from the east coast of Sabah (calculated by C H Williams Talhar & Wong). We used a discount rate of 11% per annum in our main models, used by industry in 2011 in Sabah. However, to assess model robustness we undertook sensitivity analyses using discount rates of 5%, 8% and 14% per annum. Cost data were calculated from estates past actual costs, their budgeted future costs and typical industry average costs for 2011 (obtained from C H Talhar & Williams). These costs were cross referenced and supplemented with state wide data on costs from 2008 [33]. Costs were summarised in three categories: (1) General charges (or Joint Estate costs); (2) Field upkeep (weeding; manuring; pruning; pests and disease treatment; supplying; infrastructure etc); and (3) Harvesting and transport. Costs were constant in the four models except for ‘supplying’ which is the replanting of palms if palms die. We used a commercial estates approach (rather than smallholder approach) due to data availability and use in translating industry standards to build model assumptions. In regards to ‘supplying’ in commercial estates this is undertaken within the first two years of production to ensure homogeneous age blocks [33]. Values for supplying were adjusted for the three underproductive classes to reflect the proportion of palms needed for replanting within the initial two year period (these costs were relatively small, see Tables S1 (a+b) to S4 (a+b) in File S2). Costs excluded those associated with non-recurrent costs for establishing ‘New Plantings’ and those associated with costs of palm oil processing mills.

### Modeling Oil Palm Suitability for Unprotected Forested Areas

To predict suitable and unsuitable areas for oil palm outside the protected areas, we used a Classification and Regression Tree (CART) analysis. The CART framework was developed by using data from existing oil palm blocks integrated with biophysical spatial information developed at 1 ha resolution. Predictor variables included: (1) Euclidian distance from main river (and main tributaries); (2) elevation; (3) aspect; (4) slope; and (5) a soil agriculture suitability layer using 4 suitability classes (unsuitable = 1; marginal = 2; suitable = 3; and very suitable = 4) [34]. Point training data were extracted randomly from known ‘Full stand’ and ‘Underproductive at ≤25%’ areas. ‘Full stand’ class comprised of 263 data points and ‘Underproductive at ≤25%’ had 211. We extracted raster values from the spatial predictors for each point using ArcGIS 10. Waikato Environment for Knowledge Analysis (WEKA) software was used to execute the CART analysis and a 10-fold cross-validation was used for tree pruning and the accuracy assessment. The software ArcPath was used to integrate the CART’s classification decision tree with variables to produce a categorical map of oil palm suitability. This categorical output was imported into ArcGIS 10 and extracted for the unprotected forest extent.

### Mapping Land Titles

Publicly available cadastral maps (*n* = 14) used by commercial land valuation agencies and industry were used to forecast future loss of unprotected forest by quantifying the extent of alienated land. Maps were undated but known to have been drafted in the 1990’s by the Land and Survey Department. Although, these maps have been updated since the 1990’s the extent of alienated land is likely underestimated. Maps were scanned at high resolution and geo-referenced to 2.5 m SPOT5 images. Land parcels were digitised, with title types and identity numbers recorded in a vector file.

Land title types included: (1) Native Title (NT), i.e., smallholdings alienated for agriculture (oil palm) for perpetuity and restricted to <40 ha in size [26]; (2) Country Land title (CL), i.e., ‘alienated’ State Land for commercial agriculture (oil palm) under a 99 year lease [26]; (3) Demarcated State land, with boundaries but no identity code (assumed to be under application but not alienated): and (iv) Undemarcated State land, assumed to have no title applications and not alienated (see Appendix S1 in File S1 for more details on land ordinance policies).

## Results

### Forest Extent and Forest Types

Overall classification accuracy was 72.2%, with a Kappa statistic of 0.65 using an error matrix method and 969 ground truth points (see Appendix S2 and Table S6 in File S1, for details), deemed as satisfactory e.g. kappa falls into ‘fair’ and ‘good’ according to differing scales outlined by Monserud [35] and Landis and Koch [36], respectively. Of the 520,269 ha study region, 48% (251,400 ha) is forested (inclusive of severely degraded forests) and comprised of 371 independent forest fragments (*mean* size = 679 ha; *sd* (standard deviation)  = 5,231 ha). Of the forested extent, 12% (30,173 ha) occurs outside the protected areas (i.e. unprotected forest).

**Figure 3:**
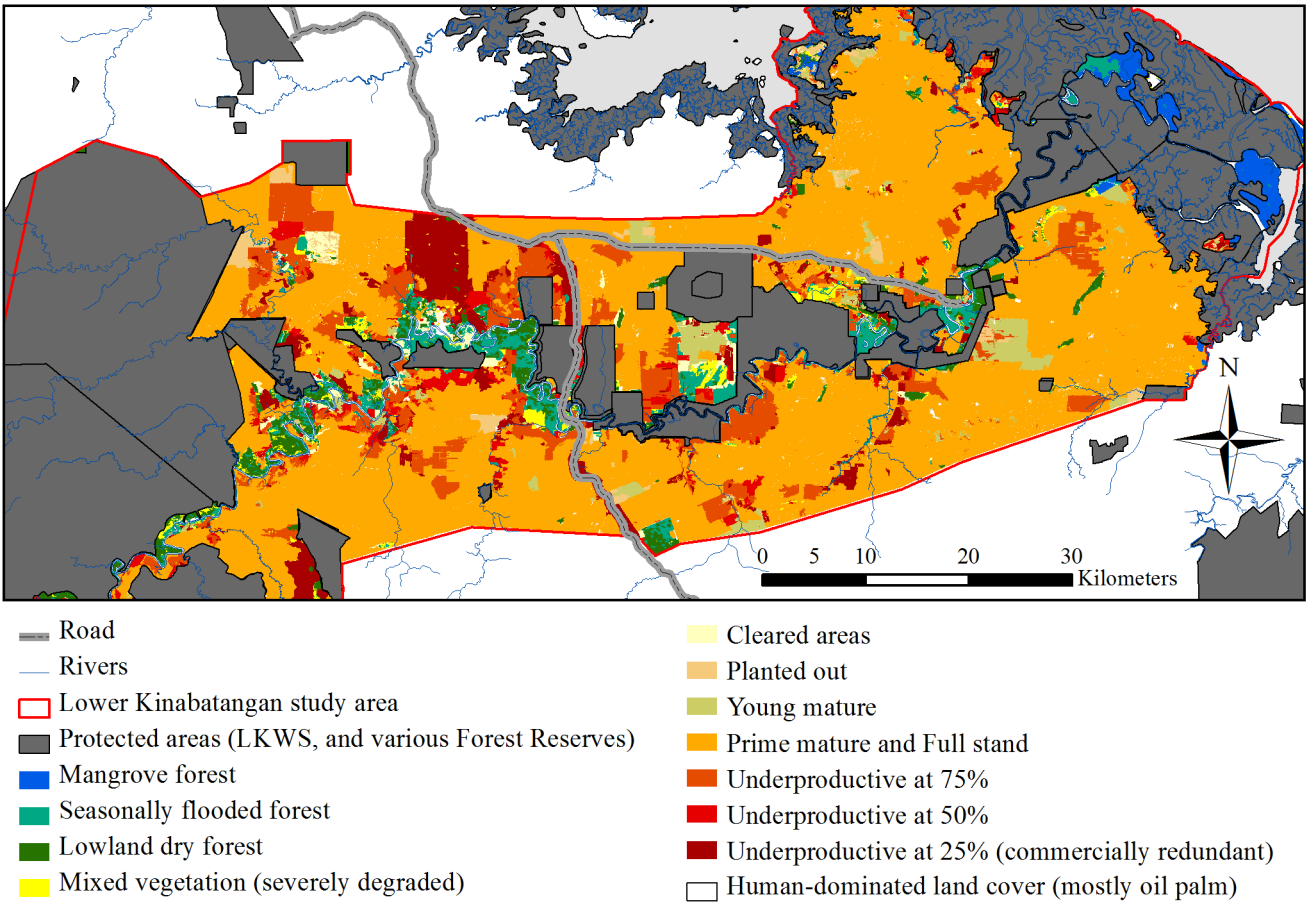
Map of forest systems and oil palm age and productivity classes. Map of the Lower Kinabatangan study area (red outline) showing the extent of the protected area network (grey) and the forest systems identified in the unprotected forest (as of 2010/2011 and generated through the Object-Based Image Analysis). Forest systems include: Mangrove forest (blue), Seasonal flooded forest (turquoise), Lowland dry forest (dark green) and Mixed vegetation that is severely degraded (yellow). Map also shows the oil palm age and productivity classes including: Cleared areas (cream), Planted out (beige), Young mature (olive), Prime mature and Full stand (orange), Underproductive at 75% (orange-red), Underproductive at 50% (red), and Underproductive at ≤25% (dark red), that are largely associated with areas proximal to the major river and its principal tributaries.

Within the unprotected forest, the Lowland dry forest system covered 32% (Table 1, Figure 3). The Seasonally flooded forest system was the largest (35%), harbouring: Freshwater swamp forest (18%); Seasonal freshwater swamp forest (14%); Peat swamp forest (<1%); and Swamp (2%) (Table 1, Figure 3). The Mangrove system comprised 19% of unprotected forest and contained: Beach forest (2%); Mangrove forest (2%); Nipah palm forest (4%); and Transitional forest (11%) (Table 1, Figure 3). These two forest systems enumerated 54% of the unprotected forest, are prone to inundation events and are likely unsuitable for oil palm. The severely degraded mixed vegetation type class totalled 14% and we predict that much of this area is prone to flooding and could also be unsuitable for oil palm.

### Future Forest Loss

Of the unprotected forest, 64% had been alienated with 49% under Native or smallholder titles (i.e., 9,497 ha, either fully or partially under 1,175 titles); and 51% under Country Land or commercial titles (i.e., 9,732 ha spanning 163 titles); the remaining extent was State land (36%) (see Table 3). Of the forest systems, 75% of the Seasonally flooded forest system had been alienated for agriculture (of this NT = 46%: CL = 54%); 21% of the Mangrove forest system (of this NT = 87%: CL = 13%); 71% for the Lowland dry forest system (of this NT = 43%: CL = 57%); and 77% for the Mixed vegetation types (of this NT = 57%: CL = 43%) (see Table 4 for more details).

**Table 3:** Land title types, size in hectares (with percentages) and number of unique demarcated titles within the Kinabatangan, classified from cadastral maps for the unprotected forests.

	Unprotected forest	
Title type	Total no. ha (%)	No. of unique titles
Native Title	9,497 (31)	1,175
Country Land Title	9,732 (32)	163
State Land Demarcated	3,937 (13)	104
State Land Un-demarcated	7,009 (23)	-
TOTAL	30,173	1,338

**Table 4:** Land title type and extent (in ha and percentages) for: Forest systems; and, Modelled oil palm suitability (CART analysis) for suitable (‘Full stand’) and unsuitable (‘Underproductive at ≤25%’) areas.

	Land Title Type			
	Nativetitle in ha (%)	Country Landtitle in ha (%)	State land (demarcated)in ha (%)	State land (un-demarcated)in ha (%)
Forest systems:				
Mangrove forest	1,016 (11)	147 (2)	321 (8)	4,179 (60)
Seasonally flooded forest	3,635 (38)	4,235 (44)	1,110 (28)	1,563 (22)
Lowland dry forest	2,934 (31)	3,917 (40)	1,747 (44)	1,017 (15)
Mixed vegetation types	1,912 (20)	1,433 (15)	758 (19)	250 (4)
Total number of ha	9,497	9,732	3,936	7,009
Oil palm suitability modelling:				
Full stand	3,665 (39)	3,952 (41)	1,723 (44)	1,003 (14)
Underproductive at ≤25%	5,832 (61)	5,780 (59)	2,213 (56)	6,006 (86)
Total number of ha	9,497	9,732	3,936	7,009

### Oil Palm Landscape

Overall classification accuracy was 84% with a Kappa statistic of 0.816 (deemed ‘very good’ [35] to ‘excellent’ [36]) using an error matrix method with 116 test data, as four fell on cloud (see Table S7 in File S1, for details). Oil palm covered 250,617 ha (48%) of the study region (see Table 5 for ha level information and Figure 3). Seventy percent of oil palm is on commercial land titles (174,698 ha). Cleared areas covered 1.3% of the oil palm landscape; Planted out covered 1.6%; and Young mature and Prime mature and full stand collectively covered 77% of the oil palm extent. For the underproductive classes, Underproductive at 75% made up 11% and of this 39% were on NT and 59% on CL. This class largely included areas of old palm (>25 years) and/or areas of water logging both of which can increase palm mortality. Underproductive at 50% totalled 3%, and spanned both NT and CL titles almost equally and were typical on slightly undulating areas or flood prone areas. Underproductive at ≤25% covered 6%, of which 28% was on NT and 66% on CL. These areas vary in age but knowledge of some areas e.g. Pontian Alljoy and Pendiros Pontian Estates, suggest conversion dates back 20 years and are largely associated with inundation.

**Table 5:** Land title type and extent (in ha and percentages) for existing oil palm age and productivity classes.

	Land Title Type			
Palm oil classes	Totalin ha (%)	Nativetitle in ha (%)	Country landtitle in ha (%)	Un-demarcatedor State land in ha (%)
Cleared areas	3,290 (1)	1,729 (2)	1,218 (1)	343 (7)
Planted out	4,030 (2)	960 (1)	2,520 (1)	550 (11)
Young & Prime mature and full stand	191,832 (77)	49,328 (70)	140,157 (80)	2,347 (47)
Underproductive at 75%	28,081 (11)	10,959 (15)	16,563 (9)	560 (11)
Underproductive at 50%	7,575 (3)	3,489 (5)	3,754 (2)	332 (7)
Underproductive at ≤25%	15,810 (6)	4,481 (6)	10,486 (6)	843 (17)
Total area	250,617	70,945	174,698	4,974

### Oil Palm Economic Models

The net present value of oil palm for each class ranged from: $413/ha^−yr^ to $637/ha^−yr^ for the ‘Full stand’ class; $179/ha^−yr^ to $403/ha^−yr^ for the Underproductive at 75% class; $-55/ha^−yr^ to $169/ha^−yr^ for the Underproductive at 50% class; and for those areas that were grossly underproductive at ≤25% had negative NPV of between $-299/ha^−yr^ to –$65/ha^−yr^ (see Table 6). We performed sensitivity analyses for varying discount rates for all NPV models (see Table 6; and Table S5 in File S2) and compared them to our 11% discounted base models. For Full stand areas, a 5% discount rate would increase NPV by approximately 100%, an 8% discount rate would seen a 40% approx increase in NPV; and around a 26% decrease in NPV under the 14% discount rate (Table 6). Similar percentage values were found for the Underproductive at 75% models (Table 6). For Underproductive at 50% areas the lower range models did not widely differ, but for the upper range models could see a 121% increase in NPV with a 5% discount rate, 48% NPV increase under an 8% discount rate; and decrease of about 33% for a discount rate of 14%. Models for the upper range of Underproductive at ≤25% were insensitive to changes in discount rates all having negative NPV (Table 6).

**Table 6:** Range values for the main oil palm Net Present Value (NPV) (US $/ha/25 years) model discounted at 11% in the four suitability classes, as well as the outputs of the sensitivity analyses with variable discount rates of 5%, 8% and 14% (showing range NPV and percentage (%) difference from the main model at 11%).

	11% discount rate	5% discount rate	8% discount rate	14% discount rate
	lowest & highest NPV	lowest & highest NPV	lowest & highest NPV	lowest & highest NPV
Full stand	$413–$637	$813–$1,252 (101%–97%)	$579–$881 (40%–38%)	$300–$471 (−27%– −26%)
Underproductive at 75%	$179–$403	$392–$812 (119%–101%)	$263–$565 (47%–40%)	$122–$293 (−32%− −27%)
Underproductive at 50%	$-55–$169	$-48–$374 (−13%–121%)	$-52–$250 (−5%–48%)	$-57–$144 (4%– −33%)
Underproductive at ≤25%	$-299–$-65	$-505–$-66 (69%-2%)	$-380 $-65 (27%–0%)	$-242–$-64 (−19%– −2%)

### Oil Palm Suitability Modelling (CART)

The CART analysis identified distance to river, elevation and soil as strong predictors for suitable and unsuitable areas for oil palm. Aspect and slope variables were not significant. Overall classification accuracy was 75% and a Kappa statistic of 0.5, deemed as fair [35] to good agreement [36] according to differing scales. The CART decision tree identified three classification rules that dictate land feature thresholds for unsuitable areas for oil palm (Figure S3 in File S1): (1) areas ≤1,504 m from river, with elevation of ≤22.4 m above sea level (ASL); and (2) if over 1504 m from a river, ≤16 m ASL and marginal soil type; and (3) if over 1,947 m from a river, on very suitable soil and between 9.9 m–14 m ASL, with the latter two likely due to areas within lower lying depressions prone to periodic flooding in some years and/or water logged areas. Of the 30,173 ha of unprotected forest, the CART model predicted 66% is unsuitable for oil palm (Figure 1(B)). Of these unsuitable lands, 59% has already been alienated for oil palm and 11% were probably under land applications, though may now be alienated (Table 4).

## Discussion

It is likely that oil palm cultivations will continue to expand at large scales throughout the world [37], as it is estimated that global demand for palm oil will double by 2020 [38]. With such expansion careful land-use planning is needed so that multiple-benefits within landscapes can be attained and issues pertaining to biodiversity and ecosystem service loss can be mitigated [39]. We employed the Kinabatangan as a ‘model system’ to investigate spatial and economic patterns of land cover in a tropical forest system.

### Heterogeneity of Oil Palm Landscapes

We considered the oil palm landscape to understand fine-scale variability of production within the wider floodplain extent to begin formulating ideas around conservation opportunity [40], [41]. Oil palm production in many fertile floodplains may deliver high profits under optimal planting conditions [17]. Our economic models for full stand areas within the region (see Figure 3) estimated NPV from $413/ha^−yr^ to $637/ha^−yr^ using an 11% discount rate (potentially increasing by 100% with an 8% discount rate, see Table 6). However, floodplains display variable topography resulting in heterogeneous suitability for oil palm cultivation (Figure 3). Our study region is characterised by areas of inundation, particularly during the monsoon season (October to March). Our results indicated that 20.5% (51,466 ha) of the total 250,617 ha of oil palm plantations are under producing. At minimum 6.3% (15,810 ha) is likely to be commercially redundant with negative NPV estimates ranging from $-299/ha^−yr^ to $-65/ha^−yr^. These areas are largely associated with flooding in low-lying areas proximal to rivers, as predicted by our CART model (Figure 1). The economic models for the lower SPH range within the Underproductive at 50% class also demonstrated negative NPV suggesting that some of these areas could also be commercially redundant (Table 6).

Although flood mitigation measures can be implemented in flood prone areas, losses from inundation within our study region have proven to be largely ineffective and very costly [42] thereby posing a financial risk to industry. For example, in the year 2000 one company experienced palm mortalities in 5,000 hectares of immature palm, with estimated financial losses of US $3 million (equivalent to US $600/ha) due to high flood water (14 m ASL) [43]. Impacts of flood related financial losses is particularly pertinent for small scale farmers who often establish plantations using formal credit, borrow money through informal arrangements, or by investing a large proportion of their savings. Failed oil palm ventures therefore represent poor return on investment for small-scale producers [44]. Larger companies with processing mills are likely to have less associated financial risk in converting flood prone land as larger plantations may have a mosaic of land suitability thereby offsetting financial impacts. Moreover, major financial risks for some companies are generally associated with fixed costs of processing mills [45]. Nevertheless, large estates have social and environmental corporate responsibilities and conserving these forests may help companies achieve these requirements.

There were a number of assumptions in our economic models for oil palm. We assumed that the variability in yield is driven by the number of SPH. However, yield may vary due to a range of factors such as soil composition and other environmental factors [12], palm strains and management practises [32], [46]; as well as pests and disease such as ganoderma [47]. In addition to factors that may affect actual yield, a number of other limitations are at play that may affect the harvesting of the fresh fruit bunches. Sabah is currently experiencing a foreign labour shortage notably of FFB harvesters, collectors and loose fruit collectors within the estates, resulting in substantial areas of oil palm remaining un-harvested [48]. Areas may also be un-harvestable if there is poor accessibility or potential health hazards such as areas with flooding. Such factors are not incorporated into our models due to a lack of data availability, as well as difficulties in up-scaling these factors to a landscape level. Nevertheless, our findings are based on sound, regional data using justifiable assumptions and provision needed values for understanding landscape economics for the region to start to formulating understanding of potential trade-offs.

### Understanding Forest System Suitability for Oil Palm

Mapping natural forest systems at the agricultural frontier is fundamental to understanding opportunities for achieving biodiversity conservation goals [49]. This is particularly pertinent in forested areas zoned for oil palm that have low expected suitability. We estimated 54% (i.e., 16,207 ha of mangrove forest and flooded forests systems combined) to 68% (i.e., 20,555 ha with the inclusion of severely degraded forest) is unsuitable for oil palm production (Table 1, Figure 3), supported by our CART results. These areas experience significant inundation either daily (Mangrove system) or annually from Monsoon floods (Seasonally flooded forest system). If these forests are converted to oil palm, it is highly likely they will mirror the commercially redundant areas. Using our most conservative unsuitable forest extent estimate (16,209 ha) we estimate at minimum 56% was already alienated under smallholder (4,651 ha) and commercial (4,382 ha) titles. In parallel to existing commercially redundant areas, future investment of smallholders or companies may see no financial gain in these areas. If these areas are converted there could be over 32,000 ha of commercially redundant areas in the Kinabatangan region.

Further loss from the unprotected forests will be a significant blow for the Lower Kinabatangan ecosystem including for lowland forest types that are becoming increasingly threatened [50], [51]. These forests provide a wide range of ecosystem function [52] and services such as above- and below- ground- carbon storage, essential in mitigating climate change [53], erosion control thereby mitigating severe runoff into the river system and flood attenuation, wildlife carrying capacity and species dispersal essential for the viability of globally threatened and endemic species found in the region [23], [24], [54], [55]. Moreover the persistence of these forests and the populations of large mega-fauna species that reside in them are crucial to the flourishing international tourism industry in the region, essential to local livelihoods. Despite the value of these forests, significant forest extent is alienated and by law should be cultivated within several years of title acquisition [26]. The large extent of alienated forest suggests many landholders have not complied with these regulations. This could be due to a number of reasons including their understanding of these forests lack of suitability for oil palm. A window of opportunity for intervention schemes results from these unconverted forests. We discuss a number of plausible intervention schemes in the context of the State of Sabah.

### State Policy/Management Interventions

For unprotected forests, the Sabah Government has commited to establishing corridors within the Kinabatangan to help promote the viability of State priority species e.g. the Bornean orangutan and Bornean elephant [56], [57], two endangered species in the Kinabatangan. As an initial step, the Sabah Government should seek to excise State land to become incorporated into the Lower Kinabatangan Wildlife Sanctuary or as Class I Forest Reserves (i.e., protected forest status). For alienated forested land conversion may be imminent. A moratorium on conversion for these areas, regardless of forest type or level of degradation, would allow time for negotiations to be undertaken between government, the oil palm industry, and local people so that opportunities to secure these forests for conservation purposes can be sought. Solutions will need to consider land holders rights (local people and companies). Lands unsuitable for cultivation are likely to incur costs that potentially outweigh revenues and could be purchased for conservation by Government or by existing land purchase schemes by NGO’s. Additionally, mechanisms such as reducing emissions from deforestation and forest degradation (REDD+) [58] may prove useful if State policies can be aligned with such mechanisms, i.e., to permit alienated land to retain standing forest (which is not permitted under current Land Ordinance Policy) [26].

For existing commercially redundant oil palm these areas could be excised to the protected area network and prioritised for restoration by either existing initiatives (e.g. HUTAN, Nestlé Project RiLeaf and MESCOT initiatives) or new reforestation projects (potentially under a carbon credit fund [59]). Companies could benefit through promoting corporate responsibility goals and securing benefits when applying for sustainable certification under the Roundtable on Sustainable Palm Oil (RSPO) [60]. Additionally, re-planting of old palm and improving yield of existing palms could help maximise returns from existing oil palm areas, offsetting need for further oil palm expansion.

Policy reform or creation of new policies could pave a more sustainable multi-use floodplain landscape. Firstly, biophysical criteria used by the State Government to demarcate areas for oil palm could be refined to account for more regional environmental constraints such as frequency and intensity of inundation. The Sabah Water Resources Enactment (1998) requires ‘river reserves’ to be retained in cultivated areas (up to 20 m from watercourse). This is rarely implemented by companies or enforced by authorities. River reserve widths are based on minimum needs to mitigate soil and bank erosion [61]. Revision of this policy to incorporate wildlife corridor needs or development of new ‘wildlife corridor’ policies should be considered. Although we strongly recommend fine-scale planning to be undertaken to promote best use of landscapes, we do suggest minimal corridor widths of 1 km for major floodplain rivers (such as the Kinabatangan which is 1.5 km wide at river mouth and 100 m wide at the most interior point of study region). This is because ‘corridors’ should be (multi) functional landscape features [62], resistant to issues such as edge effects that can compromise their long-term persistence [63]. Additionally, our findings suggests commercially redundant areas of oil palm within floodplains are largely within 1.5 km from (major) river banks, meaning that such corridors may not significantly impact industry profits.

In this study we highlighted prevalent issues pertaining to forest conversion to oil palm in a floodplain system. Our study provides an exemplary case study of how poor planning can result in unfavourable land cover whilst little serving either profitability for landholders or conservation agendas. Regional studies such as this one should be conducted for other floodplains undergoing land cover change. Greater understanding of opportunities and constraints in these landscapes is needed to promote informed trade-off decision making at multi-scale levels. As global palm oil demand increases, ensuring responsible utility of tropical landscapes is vital in synergising a balancing between agricultural and development needs with long-term biodiversity conservation and ecosystem functioning. This study has global significance beyond our study region as we hypothesis that biophysical criteria used by many governments and international agencies for targeting areas for oil palm cultivation are likely similar to those used in Sabah [64]. Revising biophysical criteria for agricultural zoning may better promote land use. Finally the long-term viability of the Kinabatangan lie in the hands of political will and landholders decisions. We hope that this study will provision information for better decision making within the context of the Kinabatangan landscape to ensure wise steps are made to secure this ecosystem long-term.

## Supporting Information

File S1 Oil palm age and productivity map validation areas (Figure S1); Land ordinance policy under land titles (Appendix S1); Operational habitat map confusion matrix (Appendix S2); Confusion matrix table of the OBIA (Table S6); Confusion matrix table of the oil palm age and productivity (Table S7); Decision Tree from the CART analysis for oil palm suitability (Figure S3).
(PDF)

File S2 Excel economic models for four oil palm classes including: Full stand 100% palm capacity (136 SPH) (Table S1(a)); Full stand at 76% palm capacity (103 SPH) (Table S1(b)); Underproductive at 75% at 75% capacity (102 SPH) (Table S2(a)); Underproductive at 75% at 51% capacity (69 SPH) (Table S2(b)); Underproductive at 50% at 50% capacity (68 SPH) (Table S3(a)); Underproductive at 50% at 26% capacity (35 SPH) (Table S3(b)); Underproductive at 25% at 25% capacity (34 SPH) (Table S4(a)); Underproductive at 25% at 0% capacity (0 SPH) (Table S4(b)); Discounted summaries (Table S5); Yield curve (Figure S2). The authors are solely responsible for the content and functionality of these materials. Queries (other than absence of the material) should be directed to the corresponding author.
(XLSX)
