# Correction: Synergies for Improving Oil Palm Production and Forest Conservation in Floodplain Landscapes

**DOI:** 10.1371/journal.pone.0106391

**Published:** 2014-08-18

**Authors:** The PLOS ONE Staff

The image for Figure 3 is incorrect. Please see the correct Figure 3 here.

**Figure 3:**
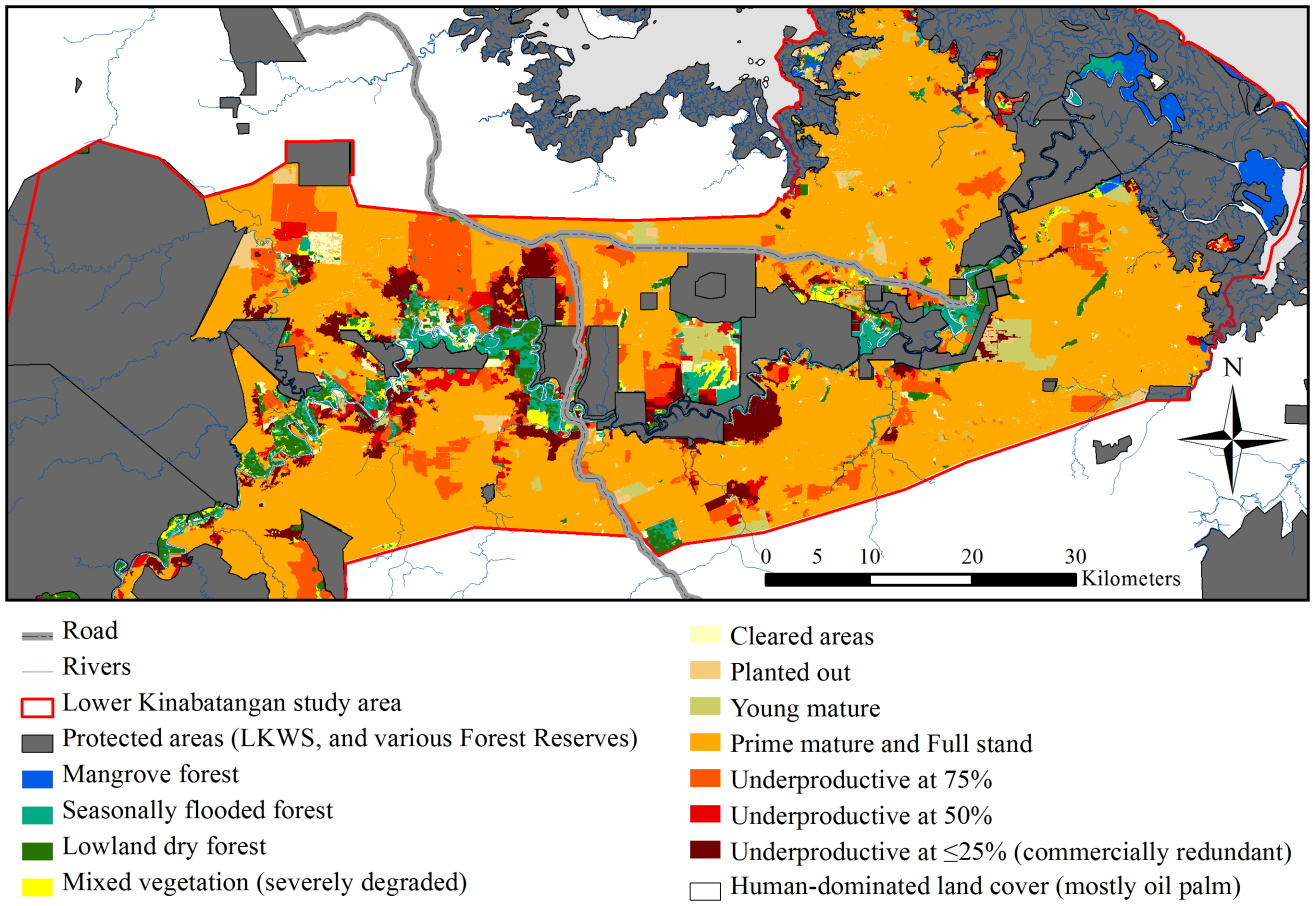
Map of forest systems and oil palm age and productivity classes. Map of the Lower Kinabatangan study area (red outline) showing the extent of the protected area network (grey) and the forest systems identified in the unprotected forest (as of 2010/2011 and generated through the Object-Based Image Analysis). Forest systems include: Mangrove forest (blue), Seasonal flooded forest (turquoise), Lowland dry forest (dark green) and Mixed vegetation that is severely degraded (yellow). Map also shows the oil palm age and productivity classes including: Cleared areas (cream), Planted out (beige), Young mature (olive), Prime mature and Full stand (orange), Underproductive at 75% (orange-red), Underproductive at 50% (red), and Underproductive at ≤25% (dark red), that are largely associated with areas proximal to the major river and its principal tributaries.
