# Supplementary material for: Synergies for Improving Oil Palm Production and Forest Conservation in Floodplain Landscapes
Source: PLoS One. 2014 Jun 2;9(6):e95388. doi: 10.1371/journal.pone.0095388 (PMC4041647; doi:10.1371/journal.pone.0095388)
Supplement: File S1 — Oil palm age and productivity map validation areas (Figure S1); Land ordinance policy under land titles (Appendix S1); Operational habitat map confusion matrix (Appendix S2); Confusion matrix table of the OBIA (Table S6); Confusion matrix table of the oil palm age and productivity (Table S7); Decision Tree from the CART analysis for oil palm suitability (Figure S3). (PDF) [file pone.0095388.s001.pdf]

File S1_Supporting information for:

Synergies for improving oil palm production and forest conservation in floodplain landscapes

Nicola K. Abram*; Panteleimon Xofis; Joseph Tzanopoulos; Douglas C. MacMillan; Marc Ancrenaz; Robin Chung; Lucy Peter; Robert Ong; Isabelle Lackman; Benoit Goossens; Laurentius Ambu; Andrew T. Knight

*Corresponding author Nicola K. Abram (nicola_abram@hotmail.com)

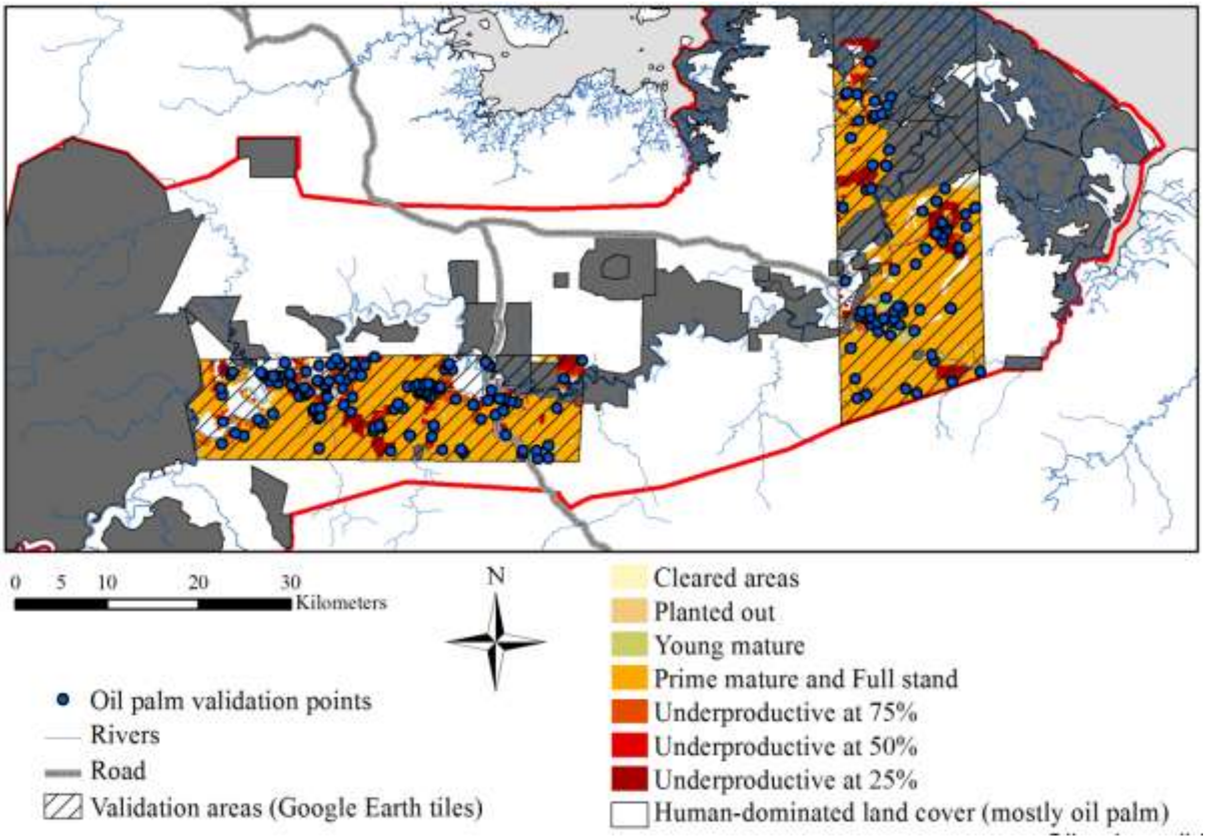

Figure S1 Oil palm age and productivity map validation areas. Map shows the Google Earth tile areas that were used for validating the Oil palm age and productivity map. The location of the randomly generated validation points (blue) are shown along with the oil palm classes (for demonstration purposes). Google Image tiles were dated 16/09/2009 (west) and 24/08/2011 (east) to best coincide with our SPOT5 2.5m resolution imagery capture dates (to minimise temporal land use differences).

Appendix S1: Land ordinance policy under land titles

State Land Ordinance Policy, see Sabah Land Ordinance, Cap. 68 (Sabah Land Ordinance ver2010), requires *bona fide* commencement of cultivation of alienated NT and CL land within six months from issue of title. Furthermore, titles ≤40 ha need to be under full cultivation within three years (Section 53.2); if between 40 ha to ≤250 ha one-fifth of the extent must be cultivated each successive year (Section 53.3); and, titles ≥250 ha equivalent yearly cultivation is compulsory until full cultivation is completed (Section 53.4). Failure to cultivate land under either CL or NT can lead to "re-entry" or seizure of lands by Government (Section 70.4) though this is rarely observed. Despite NT lands being targeted for 'native' people, clauses now permit subleasing of titles (of 30 years) to non-'native' people and companies (amended Section 17).

Appendix S2: Operational habitat map confusion matrix

Table S1 shows that four forest classes were classified perfectly (i.e., 100%). These included: Beach forest; Mangrove forest; Transitional forest; and Lowland dry dipterocarp forest. Forest types that scored ≥80% were: Nipah palm forest (94%); Freshwater swamp forest (81%); Limestone forest (80%); and the Severely degraded areas (mixed vegetation types) (80%). Swamp and Seasonal freshwater swamp forest were moderately classified at 60% and 62% respectfully (with misclassification occurring mostly with Freshwater swamp forest and Lowland dry forest) and Lowland dry forest TD demonstrated on 47% classification accuracy with confusion restricted to Freshwater swamp forest and Seasonal freshwater swamp forest. This is expected as these three forest types contain similar species (hence spectral similarity) but differ in dominant species and overall species composition.

Table S6. Confusion matrix table of the OBIA. Confusion matrix table of the merged SVM amended classification and point field training data. Codes are: Beach Forest=BF; Mangrove Forest=MF; Nipah Palm Forest=NPF; Transitional Forest=TF; Freshwater Swamp Forest=FSF; Seasonal Freshwater Swamp Forest=SFSF; Swamp=S; Lowland Dry Forest=LDF; Lowland Dry Dipterocarp Forest=LDDF; Limestone Forest=LF; and, Severely Degraded=SD. The overall accuracy of the Operational Habitat Map is 72.2%.

	**BF**	**MF**	**NPF**	**TF**	**FSF**	**SFSF**	**S**	**LDF**	**LDDF**	**LF**	**SD**	**SUM**
**BF**	17	0	0	0	0	0	0	0	0	0	0	17
**MF**	0	24	0	0	0	0	0	0	0	0	0	24
**NPF**	0	0	45	0	0	0	0	0	0	0	0	45
**TF**	0	0	0	15	0	0	0	0	0	0	0	15
**FSF**	0	0	0	0	213	64	2	56	0	2	1	337
**SFSF**	0	0	0	0	34	176	0	20	0	0	0	230
**S**	0	0	0	0	0	0	16	0	0	0	0	16
**LDF**	0	0	0	0	16	31	2	76	0	15	0	140
**LDDF**	0	0	0	0	0	0	0	0	51	0	0	51
**LF**	0	0	0	0	0	0	0	2	0	65	0	67
**SD**	0	0	0	0	0	9	0	5	0	0	2	16
**Unclassified**	0	0	3	0	0	2	7	2	0	0	0	13
**Grand**	17	24	48	15	262	282	27	161	51	82	3	969
**Correctly classified %**	**100**	**100**	**93.7**	**100**	**81.3**	**62.4**	**60.4**	**47.4**	**100**	**79.8**	**80**	

Table S7. Confusion matrix table of the oil palm age and productivity. Codes are: Cleared areas=C; Planted out=PO; Young mature=YM; Prime mature and Full stand=PM; Underproductive at 75%=U 75%; Underproductive at 50%=U 50%; and Underproductive at ≤25%=U ≤25%. The overall accuracy of the Oil Palm age and productivity map is 84%.

	**C**	**PO**	**YM**	**PM**	**U 75%**	**U 50%**	**U <25%**	**SUM**
**C**	11	1	0	0	0	0	0	12
**PO**	0	7	0	0	0	1	0	8
**YM**	0	0	18	1	0	0	0	19
**PM**	0	0	0	23	2	0	0	25
**U 75%**	0	0	0	3	14	1	0	18
**U 50%**	1	0	0	0	4	10	2	17
**U <25%**	1	0	0	0	0	1	15	17
**Grand**	13	8	18	27	20	13	17	116
**Correctly classified %**	**84.6**	**87.5**	**100.0**	**85.2**	**70.0**	**76.9**	**88.2**	

The confusion error matrix (Table S2) shows that only Young mature class was perfectly classified (100%). Cleared areas, Planted out, Prime mature and Underproductive at ≤25% had accuracies of 85%, 88%, 85% and 88%, respectively. Underproductive classes of 75% and 50% had the lowest accuracy (70% and 77%, respectively) with most confusion between these two classes.

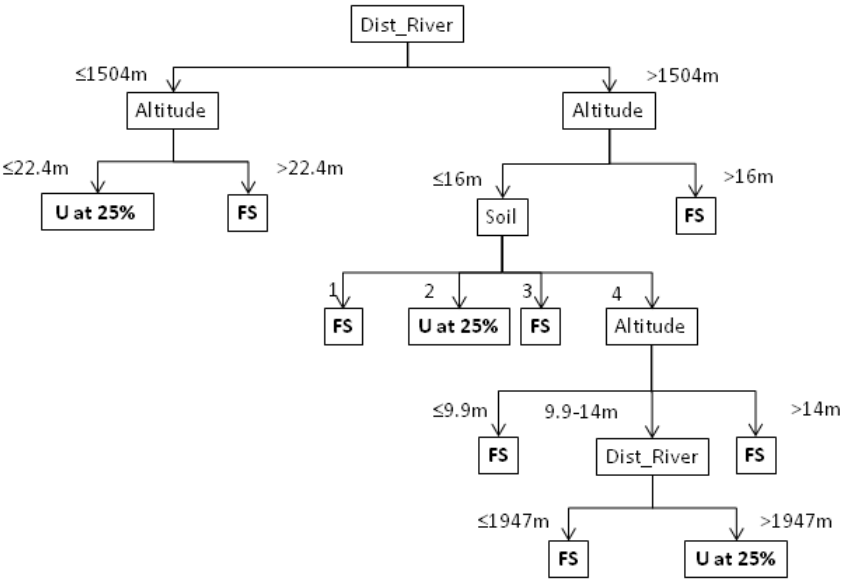

Figure S3. Decision Tree from the CART analysis for oil palm suitability. Classification and Regression Tree Analysis . Classification tree to classify Full stand (coded as 'FS') and Underproductive at ≤25% (coded as 'U at 25%') areas for palm oil within the landscape, based on the CART analysis. The classifications are: Distance from river (dist_river in meters), DEM (dem_topo in meters), and, Soil (Soil suitability classes 1=unsuitable; 2=marginal, 3=suitable, 4=very suitable). Aspect (degrees); and Slope (degrees) were incorporated into the model but were not important predictors.

References

Sabah Land Ordinance. ver2010. State of Sabah Land Ordinance (Sabah Cap.68) in L. Ordinance, editor.
